# Satisfaction and Usability of a Commercially Available Medication Adherence App (Medisafe) Among Medically Underserved Patients With Chronic Illnesses: Survey Study

**DOI:** 10.2196/63653

**Published:** 2025-01-07

**Authors:** Christa Hartch, Mary S Dietrich, B Jeanette Lancaster, Shelagh A Mulvaney, Deonni P Stolldorf

**Affiliations:** 1 School of Nursing and Health Sciences Manhattanville University Purchase, NY United States; 2 School of Nursing Vanderbilt University Nashville, TN United States; 3 Department of Biostatistics Vanderbilt University Medical Center Vanderbilt University Nashville, TN United States; 4 Sadie Heath Cabiness Professor and Dean Emerita School of Nursing University of Virginia Charlottesville, VA United States; 5 Department of Biomedical Informatics Vanderbilt University Medical Center Vanderbilt University Nashville, TN United States

**Keywords:** medication adherence, mHealth, mobile phone, app, medically underserved, chronic disease, satisfaction, usage, health disparities

## Abstract

**Background:**

Research supports the use of mobile phone apps to promote medication adherence, but the use of and satisfaction with these apps among medically underserved patients with chronic illnesses remain unclear.

**Objective:**

This study reports on the overall use of and satisfaction with a medication adherence app (Medisafe) in a medically underserved population.

**Methods:**

Medically underserved adults who received care for one or more chronic illnesses at a federally qualified health center (FQHC) were randomized to an intervention group in a larger randomized controlled trial and used the app for 1 month (n=30), after which they completed a web-based survey. Objective data on app usage were provided as secondary data by the app company.

**Results:**

The participants were very satisfied with the app, with all participants (30/30, 100%) somewhat or strongly agreeing that they would recommend the app to family and friends. Participants strongly agreed (28/30, 93%) that the reminders helped them remember to take their medications at the correct time each day, and they (28/30, 93%) found the app easy to use. Additional features accessed by some included educational features and the adherence report. Participants noted the helpfulness of having a medication list on their phones, and some used it during medication reconciliation at doctor visits. Use of the Medfriend feature, which alerts a social support person if a medication is missed, was low (n=2), but those who used it were very positive about the feature.

**Conclusions:**

A commercially available medication adherence app was found to be useful by participants, and they were satisfied with the app and the additional features provided. The use of medication adherence mobile phone apps has the potential to positively influence chronic disease management in a medically underserved population on a large scale.

**Trial Registration:**

ClinicalTrials.gov NCT05098743; https://clinicaltrials.gov/study/NCT05098743

## Introduction

### Background

Medication adherence is vital for those with chronic illnesses who require long-term medication therapies to maintain optimal health. For example, medication adherence and persistence with high blood pressure medications are known to significantly decrease the risk of both cardiac events and stroke [1,2]. In those with type 2 diabetes, medication adherence with hypoglycemics reduces microvascular complications [3]. Unfortunately, the burden of chronic diseases is increasing, with an estimated 60% of adults in the United States having 1 chronic disease and 40% having 2 or more chronic diseases [4]. The growth of chronic disease burden coupled with a lack of medication adherence is associated with increased health care expenditures due to increased demands on health care resources [5,6] and poor health outcomes such as worsening disease status and even death [5]. The economic impact of low medication adherence is estimated to cost the US health care system between US $100 billion and $290 billion annually [5-8].

Medication adherence is therefore particularly important in medically underserved populations who seek care at federally qualified health centers (FQHCs). These centers serve communities and populations with a demonstrable unmet need for health services [9]. These centers are reporting growth in more complex patient populations because their patients have higher rates of chronic conditions and social risk factors associated with poorer health outcomes [10]. Additionally, lower rates of medication adherence are seen in lower socioeconomic populations [11,12] and those with multiple chronic conditions [13]. The reasons for this are influenced by social determinants of health and the material and social conditions in which people live [14]. Adverse social determinants of health are associated with lower medication adherence [15].

Mobile health (mHealth) interventions, defined as the use of mobile wireless technologies for public health [16], have been cited as a potential way to reduce health disparities among chronically ill and medically underserved populations [17,18]. However, despite the promise of these technologies, researchers indicate that mHealth interventions remain understudied in medically underserved populations [17,18]. This is true of medication adherence apps, which can support patients in adhering to their medications through reminders, medication educational information, adherence data, and social support. Studies have shown mixed results for the interest in mobile phone interventions in vulnerable populations [18,19]. Furthermore, research testing commercially available apps to manage chronic disease in a racially and ethnically diverse sample found that the usability of the tested apps in this population was suboptimal [20]. Understanding and gathering detailed data from diverse perspectives regarding the user experience of medication adherence apps will provide important information that is needed to support wider implementation.

A recent meta-analysis of the effectiveness of mobile apps on medication adherence in adults with chronic illnesses found that medication adherence mobile apps, which are designed to be used across a range of multiple chronic health conditions, remain underexplored [21]. This meta-analysis reported that in general, patients have a high acceptance of medication apps, but none of the studies analyzed included medically underserved populations [21]. Eight studies have demonstrated increased medication adherence with the use of medication adherence apps [22-29], but only 3 of these were conducted in low-income medically underserved populations [27-29]. Two of those studies in underserved populations were focused on hypertension [27,28], and the other included hypertension and type 2 diabetes and was a post hoc analysis of a digital health offering using a cluster-randomized design [29]. Only 1 of these studies, conducted in an urban low-income population with hypertension, obtained satisfaction information on the intervention [28]. Satisfaction with the app was high, and most participants felt they would use the app or a similar program in the future. Participants agreed that the app made it easier to keep track of their medications and that having a medication list on their phone made it easier to take care of themselves. More detailed feedback from the participants or information on which features of the app were used was not gathered [28].

A high-quality, free, commercially available smartphone medication adherence app called Medisafe supports patients in adhering to their medication regimen across disease states [30]. It uses a variety of advanced features, such as daily reminders, which can be snoozed, rescheduled, and marked as taken or missed; medication educational information in the form of medication cards and videos; an interaction checker; customizable refill reminders; adherence reports; and the ability to designate a social support person to be notified if a medication is skipped [23]. A randomized controlled trial (RCT) mixed methods evaluation in patients with coronary artery disease examined the efficacy [23] and the utility, acceptability, and engagement [31] of the Medisafe app. This study was conducted in a large urban tertiary hospital in Sydney, Australia and did not focus on a medically underserved population. In addition to improving self-reported medication adherence, overall utility was rated positively, with participants indicating that having their medication list on their phone and receiving timed reminders were useful. Most participants engaged with the app and its features; found the app acceptable, convenient, and easy to use; planned to continue using the app; and would recommend it to a family member or friend [31].

A qualitative study explored the potential benefits and barriers of using a mobile medication app in a medically underserved population in the United States [18]. The researchers found that patients were willing to try smartphone apps but expressed concerns about affordability, the technology being too complicated, not keeping phones with them all the time, and not being able to use all the features [18]. That study exposed a knowledge gap regarding the perceptions and user experiences of medically underserved patients with chronic illnesses who use free commercially available medication adherence apps.

### Purpose

To address the knowledge gaps, a larger RCT investigating the efficacy of the Medisafe app (reported elsewhere) [32] was performed for evaluating the overall use and satisfaction of patients with a variety of chronic illnesses in a medically underserved population in an FQHC in the United States. The efficacy portion of the RCT found significant improvements in both medication adherence (Cohen *d*=0.52; *P*=.01) and medication self-efficacy (Cohen *d*=0.43; *P*=.04) for participants assigned to use the app compared to the usual care group [32]. As part of this RCT, participants assigned to the intervention arm provided feedback and usage data regarding their experience using the Medisafe app [32]. This manuscript presents the summaries of the perceptions of patients enrolled in the intervention arm of the RCT regarding the usefulness of and satisfaction with the app features after 1 month of use. Given the improvements observed in medication adherence and self-efficacy, understanding patients perceived usefulness and satisfaction with the app is important to address potential barriers for uptake and use in larger medically underserved patient populations who often receive care for chronic illnesses in FQHCs.

## Methods

### Setting and Recruitment

Participants were recruited from November 2021 through June 2022 from an outpatient adult medicine department in an FQHC in the northeastern United States. The inclusion criteria for the RCT study were as follows: (1) adults aged 18 years or older, (2) having the ability to speak and understand English, (3) personally owning and using an Android smartphone (version 5.0 or above and at least 88 MB of phone space) or iOS smartphone (version 13 or later and at least 165 MB of phone space), and (4) taking at least one medication for a chronic condition based on the computerized medical record at the health center. Patients were excluded if they: (1) were already using a medication reminder app or other electronic reminder system such as phone alarms, (2) owned a smartphone not capable of downloading the app, (3) had a diagnosis of severe dementia or serious mental illness, or (4) were otherwise unable to use a mobile phone or the medication reminder software either physically or cognitively. For this study, only those participants who were randomized to the intervention group and used the Medisafe app were invited to participate in the survey.

Recruitment involved an informational flyer, a referral form from clinicians at the health center, and in-person recruitment. The flyer and referral forms were available to clinicians, staff, and patients in the FQHC offices and at the reception desk. The form contained study information, the contact information of the principal investigator (PI), and a place for patients interested in participation to provide their contact information. The form also contained a section for health care providers (HCPs) to refer potential patients and a section for their signature to verify that the patient’s medications listed in the electronic health record were correct and current. The PI (CH) conducted in-person recruitment at the FQHC on multiple days per week and worked with clinic staff to identify potentially eligible patients. Although a convenience sample was used (ie, patients visiting the clinic on any given day), the risk of selection bias was reduced by using the aforementioned 2-prong approach to identify eligible patients for recruitment, inviting all patients meeting the eligibility criteria to participate, and using random assignment to either the intervention or control group. The 2 groups were not statistically different [32]. The PI approached eligible patients at the end of the health center visit to inform them of the study. Once the PI confirmed participant eligibility and obtained informed consent, participants were randomized to either the intervention or control group. Additional details of study procedures for the RCT have been previously published [32].

### Statistical Analysis

Based on a preliminary efficacy study for the RCT [33], a total sample of 60 participants was estimated to enable the detection of differences between the groups with Cohen *d* effect values of 0.6-0.7 (80% power; α=.05) for the quantitative study variables [32]. As 30 participants were randomized to the app intervention group, their usage and satisfaction data are presented in this manuscript. Descriptive statistics and frequency distributions were used to describe the sample and determine if the data were normally distributed. Qualitative participant responses were transcribed into an Excel spreadsheet, and the content was coded and summarized as themes by the researcher (CH) and PhD faculty advisor (DPS).

### Ethical Considerations

This research was approved by the Vanderbilt University Institutional Review Board (IRB #211409) and is registered with ClinicalTrials.gov (NCT05098743). All participants received a copy of the consent form. Based on participant preference, informed consent was completed as either an IRB-approved e-consent form or a hard copy. The consent form contained a privacy and confidentiality protection description ensuring that all study data are deidentified. Participants received a US $25 gift card after completion of the baseline survey and a US $35 gift card after completion of the follow-up survey.

### Medisafe App Intervention

The Medisafe app is a Health Insurance Portability and Accountability Act (HIPAA) compliant medication adherence app that is available at no cost in the iTunes and Google app stores. In previous studies, Medisafe was ranked highly among medication reminder apps [30,34]. The Medisafe app provides interactive and customizable daily timed reminders to reinforce medication taking at a set time every day through a push notification, equivalent to an alarm or text message. The reminders can be snoozed, rescheduled, or marked as taken or skipped, and they are repeated a total of 3 times in 10-minute intervals if the participant does not mark the medication dose. Additional features include educational information in the form of a medication database that includes written and video content [30]. The written content is in the form of a medication card, which Medisafe terms a leaflet, and it reviews what the medicine is used for, medication interactions, what to do if the user misses a dose, what the user should watch for, possible side effects, how it should be used, and where to store the medication. Some medications also have video content, consisting of a brief clip of an HCP reviewing the most important considerations when taking the medication, which can be viewed on tapping the information icon. There is also an interactions tab that lists possible interactions with the medications or food/alcohol. Lastly, there is an interaction checker where participants can check for interactions between their medications. The app also has a Medfriend feature, which allows participants to designate a family member or friend as their support person. The Medfriend feature will alert the designated Medfriend who can provide peer support and additional reminders through text messages, emails, or a telephone call if the patient misses a dose. The language mode of the app can be switched, if desired, to multiple foreign languages, including Spanish.

The PI helped consented participants set up the app using a copy of the patient’s medication list extracted from the electronic health record. The PI also reviewed how participants could access and edit their medications; access medication educational content; and indicate when a medication was taken, skipped, or rescheduled. The PI reviewed with participants additional app features such as Medfriend, medication interaction checking, adherence reports, and refill reminders. Participants were also shown how to access the help and support section in the app. Following the app set up, the PI provided previously developed educational materials as a take-home resource. These materials, specific to either an iPhone or Android smartphone, included a laminated “quick tips” card with short instructions on the reviewed features and how to access them. Additional detailed instructions on how to use the app were printed in a question-and-answer format and distributed to participants.

### Data Collection and Study Procedures

All study data were collected using observation, a REDCap web-based survey, and secondary data provided by the app company and were obtained using a data-sharing agreement between institutions. REDCap is a secure web-based software platform designed to support data capture for research studies [35,36]. Following consent, all participants completed the baseline study survey. Those randomized to the intervention group also completed a survey at study end to obtain feedback on the app, including usability and satisfaction.

### Observation

While setting up the app for the intervention group, the PI completed a study-specific observational behavioral checklist. The purpose of the checklist was to inform the researchers if participants had difficulty setting up the app and how long it took them to do so. The checklist included documenting whether the participant had difficulty visualizing the app and had difficulty with dexterity while setting up the app, and mentioning the number of times the participant’s input of medications needed to be corrected. The length of time in minutes from starting the download of the app to completing app set up and reviewing the app was also documented.

### Survey

After 1 month, based on preference, participants completed the follow-up survey online, by phone, or in-person at the health center. Participants who did not complete the follow-up survey within 10 days of the 1-month follow-up date received 2 reminders via phone, email, or text message.

### Measures

The end-of-study survey included 11 questions that assessed satisfaction and usability using a 5-point Likert scale with responses ranging from “strongly disagree” to “strongly agree.” The survey questions were developed and pilot tested before use [33]. Seven of the first 11 questions were developed by Santo et al [31] and were used with permission in this study, while the remaining 4 were developed by the researchers. Six additional questions asked about the use of additional features such as the educational information, Medfriend feature, interaction checker, adherence report, refill reminder, and additional morning reminder of the Medisafe app. These questions asked participants whether they used a given feature, and if they did, whether they found the feature useful. There were open-text response options available to elicit qualitative data from the app participants such as how a feature helped them manage their medications and what they found most useful about the feature. The remainder of the survey included 6 general use questions previously developed and pilot tested, 4 of which were “Yes/No” questions (eg, did you use the refill reminder and did you have technical issues with the smartphone app?). The remaining 2 questions assessed how often medication reminders were received each day and which language the participant used.

### Secondary Data

Deidentified usage data were obtained from the Medisafe company at study completion. Medisafe provided the PI with objective user interactions with the Medisafe app, such as whether educational information in the form of a leaflet was accessed by participants and whether the Medfriend feature was used.

### Analysis

Descriptive statistics and frequency distributions were used to describe the sample and determine if the data were normally distributed. Open-ended survey question responses were imported into an Excel (Microsoft Corp) spreadsheet, and the content was coded and summarized as themes by the researcher (CH) and the PhD faculty advisor (DPS). This approach was taken given the short, free-text, and limited responses received. Since the qualitative data came from the open-ended survey responses, data collection was based on sample size rather than data saturation.

## Results

### Participant Characteristics

**Figure 1:**
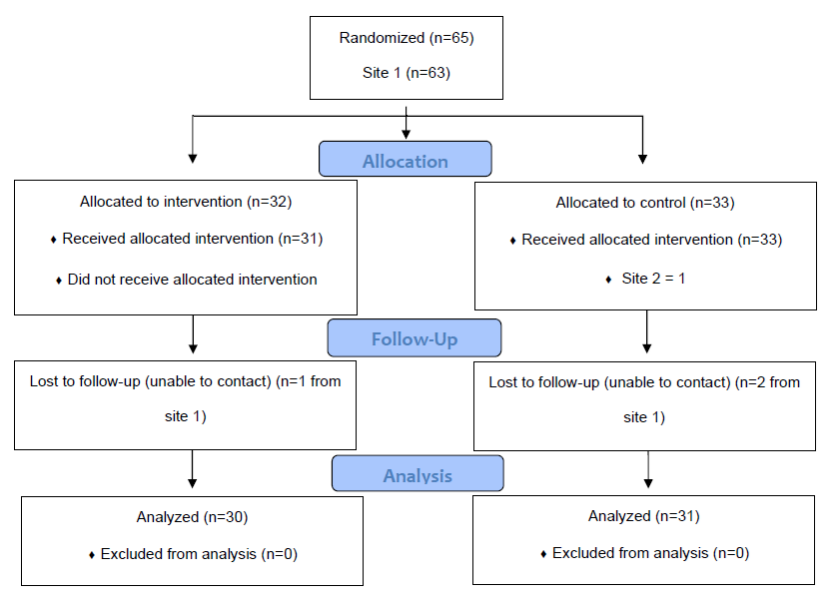
Flowchart of study participants in the larger randomized controlled trial of the efficacy of the app intervention.

Complete details are included in Table 1, and information can also be found in the app efficacy manuscript [32]. A flowchart of study participants in the main RCT can be found in Figure 1. The median age of the 30 participants using the app was 53.5 years (IQR 37-76 years). Most participants in the intervention group were non-White (23/29, 79%). Races/ethnicities were as follows: Asian (5/29, 17%), Black or African American (10/29, 35%), Hispanic/Latino (4/29, 14%), Native American or Alaska Native (1/29, 3%), and other (3/29, 10%). More than 75% of the participants had government insurance (25/30, 83%), and a small number of participants were uninsured (2/30, 7%). Brief health literacy scores were generally high (median 12.0 of a possible score of 15, IQR 5-15). Slightly more than half of the participants (16/29, 55%) reported that it was either very or somewhat difficult to pay their monthly bills. The most common chronic illness was hypertension (22/30, 73%), followed by hyperlipidemia (19/30, 63%) and type 2 diabetes (14/30, 47%). Most participants (25/30, 83.3%) had 2 or more chronic illnesses.

**Table 1:** Sociodemographic characteristics of the participants using the app.

Characteristic		Value, n (%)
**Race/ethnicity (N=29)**		
	Asian	5 (17)
	Black or African American	10 (35)
	Hispanic/Latino	4 (14)
	Native American or Alaska Native	1 (3)
	White	6 (21)
	Other	3 (10)
**Marital status (N=30)**		
	Married/partnered	15 (50)
	Single/never married	15 (50)
**Employment status (N=30)**		
	Employed	15 (50)
	Unemployed	12 (40)
	Retired	3 (10)
**Education (N=30)**		
	Some high school or less	8 (27)
	High school graduate	4 (13)
	College credit, no degree	8 (27)
	Trade/vocational training	4 (13)
	Associate’s degree	2 (7)
	Bachelor’s degree or higher	4 (13)
**Difficulty paying bills (N=29)**		
	Very difficult	6 (21)
	Somewhat difficult	10 (35)
	Not very difficult	8 (28)
	Not at all difficult	5 (17)
**Type of health insurance (N=30)**		
	Uninsured (sliding scale)	2 (7)
	Government insurance	25 (83)
	Private insurance	3 (10)
**Current chronic illness (N=30)**		
	Hypertension	22 (73)
	Type 2 diabetes	14 (47)
	Hyperlipidemia	19 (63)
	Asthma	5 (17)
	Other^a^	11 (37)

^a^Includes depression (n=1), type 1 diabetes (n=1), chronic obstructive pulmonary disease (n=1), heart disease (n=1), cirrhosis (n=1), anxiety (n=1), gout (n=1), rheumatoid arthritis (n=1), thyroid disorder (n=1), hypothyroidism (n=2), gastroesophageal reflux disease (n=1), arthritis (chronic pain) (n=1), and fibromyalgia (n=1).

### Behavioral Observations While Setting Up the App

During app set up, some participants (4/30, 13%) expressed difficulty visualizing the app owing to the unavailability of their eyeglasses, which they stated were either in their car or left at home. No participants had difficulty with dexterity while setting up the app, and the median time it took from starting the app download to completing the set up and reviewing the app was 15 minutes (IQR 10.0-25.0; minimum 10, maximum 30 minutes). The majority of participants (21/30, 70%) did not need to be corrected when they entered the medications. However, 4 (13%) were corrected by the researcher once, 3 (10%) were corrected twice, and 2 (7%) were corrected thrice. Patients were corrected when they spelled the medication name incorrectly, chose the incorrect medication dose, or set an incorrect time for the reminder.

### Satisfaction and Utility

Summaries of the participants’ reports of satisfaction are presented in Table 2.

Most participants (27/30, 90%) strongly agreed that they liked the app design, while most (25/30, 83%) strongly agreed that it was useful to have their medication list on their smartphone. Some participants (2/30, 7%) mentioned the usefulness of the app when seeing other HCPs to indicate the medications they were taking during medication reconciliation. Furthermore, a large proportion of participants (28/30, 93%) strongly agreed that the reminders helped them to remember to take their medications at the correct time each day. The majority of participants strongly agreed that the app was easy to use (27/30, 90%) and convenient (28/30, 93%) and that they would continue using the app (26/30, 87%). A small number of participants (2/30, 7%) somewhat agreed that they would continue using the app. It is important to note that some participants (2/30, 7%) strongly disagreed that they would continue using the app, because they found the reminders annoying. All the participants (30/30, 100%) somewhat agreed or strongly agreed that they would recommend the app to family and friends.

**Table 2:** Satisfaction with the app (N=30).

Satisfaction information		Value (N=30), n (%)
**Liked the app design**		
	Neutral	1 (3)
	Somewhat agree	2 (7)
	Strongly agree	27 (90)
**It is easy to tap the correct icon with my finger**		
	Neutral	1 (3)
	Somewhat agree	1 (3)
	Strongly agree	28 (93)
**I am able to see all the options in the app**		
	Neutral	3 (10)
	Somewhat agree	1 (3)
	Strongly agree	26 (87)
**It is useful to have a medication list on the smartphone**		
	Somewhat disagree	1 (3)
	Neutral	1 (3)
	Somewhat agree	3 (10)
	Strongly agree	25 (83)
**Reminders helped me remember to take my medications at the correct time each day**		
	Neutral	1 (3)
	Somewhat agree	1 (3)
	Strongly agree	28 (93)
**Found it easy to use the app**		
	Somewhat disagree	1 (3)
	Neutral	1 (3)
	Somewhat agree	1 (3)
	Strongly agree	27 (90)
**Found it easy to set up reminders in the app**		
	Neutral	3 (10)
	Somewhat agree	2 (7)
	Strongly agree	25 (83)
**Found it convenient to have the app**		
	Strongly disagree	1 (3)
	Somewhat disagree	1 (3)
	Strongly agree	28 (93)
**Found it useful to snooze the reminder**		
	Strongly disagree	1 (3)
	Somewhat disagree	1 (3)
	Neutral	10 (33)
	Somewhat agree	3 (10)
	Strongly agree	15 (50)
**Will continue using the app**		
	Strongly disagree	2 (7)
	Somewhat disagree	1 (3)
	Neutral	1 (3)
	Strongly agree	26 (87)
**Will recommend the app to family and friends**		
	Somewhat agree	2 (7)
	Strongly agree	28 (93)

The 2 participants (10%) who were dissatisfied with the app described the reasons why. One said:

It is annoying when you get a reminder and you are in the middle of doing your work. This is not for everybody. I work on the computer and on my cell phone and it is very distracting to receive the reminder in the middle of working on something. It might be better for someone who doesn't have as much going on. I find it very distracting.

The other participant had technical difficulties but blamed it on the type of phone they had:

The bug thing with the notification alarms was a problem. I have an android - a cheap phone. My phones get destroyed because of the type of work I do.

### Use of Educational Information

Almost half of the participants (12/30, 40%), self-reported accessing educational information. To the contrary, objective usage data from Medisafe indicated that only 3 participants (10%) accessed the educational content, which was defined as cards termed “leaflets” or videos. According to Medisafe, this was done for a total of 14 medications. Additionally, Medisafe reported that only 1 participant accessed 4 different videos and 1 leaflet, 1 participant accessed 1 leaflet, and 1 participant accessed 6 different videos and 2 leaflets. Although not all participants actually accessed the information, those who reported accessing the educational information (12/12, 100%) found the information useful. When asked about how they used the educational information in the app, the participants reported learning about the side effects of the medications (6/12, 50%), reported that it was helpful for general knowledge (4/12, 33%), and mentioned using it to learn more about medication and food interactions (2/12, 17%).

### Medfriend Feature

Based on usage data from Medisafe, only 1 participant (3%) used the Medfriend feature. That participant was very positive about the feature and reported that her husband would call her to say, “Are you taking your medicines?” She stated:

It gets him involved. It makes him recognize that I need support and I need to take the medicine. It makes me know he loves me.

Another participant self-reported using the Medfriend feature, but there was no indication of use in the Medisafe data. The participant reported that when her husband was notified, he would send a text about her forgetting her medications and she would remember to take them. Those who did not use the Medfriend option were asked, “who might that person be for you?” Among those who responded (18/30, 60%), the top 3 most common responses were their sibling (4/18, 22%), their child (4/18, 22%), and their husband or wife (4/18, 22%).

### Interaction Checker

All participants who used the interaction checker (5/30, 17%) agreed that it was useful. One participant reported that 2 of the medications she had been taking together should be taken separately and stated, “It was a lifesaver!” The other 4 participants expressed an appreciation for being able to have access to this type of information. The use of the interaction checker was distinct from the educational content and could not be verified in the Medisafe data as Medisafe does not register or track the use of the interaction checker.

### Adherence Reports and Reminders

Participants (8/30, 27%) who checked their adherence report agreed it was useful. The adherence report provided them with a history of their daily missed and taken medications as well as a weekly adherence percentage based on what they reported when marking medication reminders in the app. Participants reported experiencing positive reinforcement for adhering to their medications through the adherence report, mentioned the affirmation they received when they saw a high percentage of adherence, and reported appreciating the positive reinforcement as useful. Some participants (2/30, 7%) mentioned that it incentivized them to reach higher levels of adherence.

Slightly less than half of the participants (13/30, 43%) received reminders to take their medications 2 times a day, while around one-third (9/30, 30%) received reminders 3 or more times a day. Reminders were generally well received:

I like the reminder. The shaking of the pill bottle helps me. Sometimes I will wake up at night and remember hearing the shaking pill bottle that day and I will get out of bed and check if I took my pills that day. I might be cooking with the grandkids and the first reminder goes off. I might ignore it but with the second reminder I might put the bottles on the counter so I can remember.

Three participants (15%) mentioned the app’s helpfulness, particularly for those who have multiple chronic illnesses and take multiple medications.

This app was a lifesaver. I take a lot of different medication so sometimes I forget whether I took the medication or not. I can check the app to see if I took it or not.

One of those 3 participants commented as follows:

It is perfect for people who have multiple illnesses and take a lot of different medications. I take eight different medications a day.

Most of the participants (27/30, 90%) did not use the refill reminder. In their comments, a number of participants said they received automatic refill reminders from their pharmacy and therefore did not need this feature of the app. All participants (30/30, 100%) used the app in English, and the majority (24/30, 80%) did not make any changes, such as changing the time of a reminder, removing or adding a medication, or changing the medication dose in the app. Many participants (13/30, 43%) said they would use the app to manage someone else’s medications.

### Technical Difficulties Using the App

Some participants (4/30, 13%) mentioned they had technical difficulties. Of those who reported difficulties, 3 (75%) needed to allow notifications from the app to hear the reminders. Another user mentioned that they had to tap the “take all” icon a number of times before it registered and suggested that it should be made bigger or be more centrally placed. Participants gave additional feedback about the app when asked (20/30, 67%). In this section, participants (7/20, 35%) specifically mentioned liking the reminder.

It was really nice to hear that shaking sound. It was fun.

Some participants (2/20, 10%) reported that the snooze function was particularly helpful when they were away from home.

The snooze option is helpful to use when I am out and don’t have my medications. When I come back home it reminds me so I remember to take it.

### Social Support

The 2 participants (7%) who self-reported using the Medfriend feature were very enthusiastic.

It's a great app and I love it. My husband is on it for his meds and I am his Medfriend. I am also going to get my mother hooked up on it.

The other participant shared her thoughts about the feature, highlighting her increased feelings of self-efficacy and social support.

This app is about being a team player. You are able to help me and I am able to help you. I can now say “I did it” “I can do this.” This is a good app. I can't see anyone who is interested in their health not using this app. Since being introduced to this app I know that it is there for me.

## Discussion

### Principal Findings

As part of an RCT using the Medisafe medication adherence app in a medically underserved population with a variety of chronic illnesses, behavioral observations on app use and satisfaction and usage data were gathered from participants in the intervention arm of the study and the Medisafe company. The quantitative RCT results (reported elsewhere) [32] found significant improvements in both medication adherence and medication self-efficacy for participants who used the app. The portion of the RCT presented in this manuscript, which collected behavioral observations and satisfaction and usage data from the intervention arm, identified that participants were satisfied with the app and found it useful. Even though the use of the additional features was generally low, those who used them found them useful. Most participants did not need help setting up the app. An important strength of this RCT is that it explored patients’ perceptions of the usefulness of the app and their satisfaction with the app and therefore fills an important knowledge gap. This was done by collecting both quantitative and qualitative data through open-response questions, which gave voice to the perspectives of a low-income racially and ethnically diverse sample of adults with chronic illnesses receiving care in an FQHC [17,18]. As FQHCs are reporting a growth in the rates of treating complex chronic conditions [10] and there are lower rates of medication adherence among populations with lower socioeconomic status and those with multiple conditions [37], the implementation of tools to enhance medication adherence is imperative. Understanding the user experience with the Medisafe mobile app demonstrated that wider-scale use of the Medisafe app is feasible in a low-income population with multiple chronic illnesses. Systematic literature reviews have pointed out a gap in implementation studies of mobile app interventions in this population [38]. This study addressed an important knowledge gap by demonstrating that the use of a commercially available free medication adherence app is a viable option for medically underserved adult patients with chronic diseases.

Prior research found that medically underserved patients expressed reluctance about paying for a medication adherence app [18]. While not directly addressed in this study, some participants anecdotally asked before enrolling in the study if they would need to pay for the app, and when told it was free, they expressed interest in participating. This underscores the importance of not having patients incur additional costs for the technology and was a strength of this intervention.

Most participants were able to set up the app with minimal assistance, with a median duration time of 15 minutes during the behavioral observation. It is important to note that 30% of the participants needed to be corrected 1-3 times when setting up the app, thus pointing to the importance of helping some patients set up the app initially and checking that the medications are entered accurately. This highlights a difficulty with individuals setting up the app. Although not implemented in this study, another option is to import medications from other databases, such as Apple Health, or a pharmacy directly. This may shorten the time it takes to set up the app. Once the app is set up properly, in addition to HCPs assisting patients, the help and support page and the company contact could serve as a resource for patients.

Survey data indicated that satisfaction with the app was high, with most patients strongly agreeing it was easy to use. All intervention group participants (30/30, 100%) strongly agreed that they would recommend the app to family or friends. This was higher than the proportion in the study by Santo et al [31], which used Medisafe and found that 78.6% of patients with coronary heart disease would recommend it [31], and the study by Anglada-Martinez et al [24], which used a similar app and found that 71.4% of patients receiving treatment for hypertension, dyslipidemia, heart failure, or HIV would recommend it [24]. Both these studies were, however, conducted outside of the United States. It may be that Americans are more familiar with app technologies and feel more comfortable recommending apps to others.

Qualitative research conducted in a medically underserved population with chronic illness regarding the use of medication adherence apps found that technical issues and complexity were concerns when setting up and using these apps [18]. One study involving a medication adherence app similar to Medisafe indicated that 50% of participants reported problems receiving reminders [24]. The Medisafe app used in this study is a commercially available app with high-quality assessment ratings [30,39], and in this study, technical issues were rare. The most common issue was not receiving the reminders until the participant allowed notifications from the app in their phone settings. No participants expressed that the app was technically complex, which was previously cited as a concern in this population but was not an issue in this study [18]. One participant had ongoing technical issues receiving reminders. These findings suggest that the Medisafe app can be implemented in this population from a technology standpoint, and participants did not find it difficult to use.

Similar to other studies involving the Medisafe app, feedback results point to receiving timed medication reminders as the most used aspect of the app [31]. Furthermore, feedback regarding the app aligns with the findings of other studies linking medication reminders with medication adherence [22,25,28,29]. Participants found the snooze function of the reminder helpful when they were not home to take their medications and used this function as a reminder to take their medications when they got home. The snooze function therefore was an important component of the app when participants experienced disruptions in their routines, such as being away from their medications. The findings also align with a previous study in a medically underserved population where participants indicated that disruptions in their daily routines negatively affected their medication adherence [18]. This study demonstrated that patients used the reminder feature when available, and the majority of patients found it helpful in improving medication adherence. The 1 participant who was bothered by the reminders used his phone for work and found the reminders distracting if he was using his phone for work purposes. The reminders predominantly targeted the phenomenon of forgetting, which has been found by a study to be the most likely cause of reported nonadherence in low-income uninsured patients with multiple chronic illnesses [13].

Research has shown that both patient knowledge of medications and their satisfaction with the information provided about their medications can improve medication adherence [40]. There was a discrepancy between the data reported by Medisafe and the number of participants who self-reported accessing educational information. Although the reason for this discrepancy is not clear, several possibilities exist. First, participants could have overstated the use of educational features. Another potential reason might be related to the specific data Medisafe defines as educational data. Medisafe does not collect data on the use of the interaction tracker or the “For You” tab at the bottom of the app and only collects data if a participant clicks on the educational leaflet and opens it up. In contrast, participants might have perceived content under the “For You” tab and drug interaction materials or videos as educational materials, resulting in a discrepancy between patient self-reported data and Medisafe data regarding accessing educational content. The feedback received demonstrated that participants who reported accessing the educational information (less than half) were very positive about doing so. The educational information was found to be useful for learning about side effects and food and medication interactions. Because individuals have different preferences for the amount of medication information they receive and the way that information is delivered [41], the modularity of the Medisafe app is useful to facilitate patient education in a practical and less burdensome way. The information is available at the patient’s fingertips and can be accessed as frequently as needed to learn what they want at their convenience. The educational app feature is also advantageous to HCPs as it alleviates some of the burden and time commitment associated with educating patients about their medications.

Social support has been found to have a positive effect on medication adherence [42-44]. Studies deploying digital technologies in the form of web-based online communities to provide social support have generally demonstrated that they can support people emotionally, socially, practically, and politically [45]. However, using technology to provide social support has not been studied in the context of a commercially available medication adherence mobile app. This study addressed this gap by studying social support via a commercially available app in the context of medication adherence. The Medisafe app offers social support in the form of Medfriend, and this is the first known study to incorporate this feature as part of the study intervention. Some studies have pointed out that online social support networks for those with specific chronic illnesses lessened the burden on relationships with family and friends, who are referred to as “offline” support persons [45,46]. However, despite asking and offering to demonstrate how to set up the Medfriend feature, usage of the Medfriend feature was very low. This study did not gather information on why participants chose not to set up the Medfriend feature, and this is a limitation of the study. It may have been because this feature was seen as too burdensome by the patients or their support people, most of whom were identified as family members. Patients might have avoided using Medfriend due to confidentiality concerns associated with this feature, which entails giving access to the user’s medication list, as many participants (13/30, 43%) were willing to use the app to manage someone else’s medications but chose not to share their own medication information. Another challenge regarding the Medfriend feature was that there was a discrepancy between Medisafe data and self-reported data on the use of the Medfriend feature. Despite the aforementioned concerns, the 2 participants who reported using the Medfriend feature were satisfied with it as they perceived that the app fostered social support. To further explore the social support feature of the app, research on dyads who use the app to manage the medications of family members might shed new light on the phenomenon of incorporating social support into mobile apps. By studying a subset of the population, including patients and their caregivers, the social support feature may be used more frequently. If the confidentiality of medication lists proves to be a barrier, a feature that dissociates specific medications from the reminder might address that concern. Support persons could receive a general text that their online Medfriend has not taken their medications without sharing details on the specific medications.

Participants reported that having a list of medications on their phone was beneficial, which was also noted in a population of patients with coronary heart disease who used the Medisafe app in Australia [31]. When managing chronic illnesses, patients are often referred outside of the FQHC setting or require hospitalization to receive care. Some participants mentioned using the phone medication list for medication reconciliation when seeing other HCPs. This finding is in contrast to that of another study of patients presenting to an emergency department setting, which found that emergency department patients rarely used their mobile phones to share their medication list during medication reconciliation [47]. Medication reconciliation can be facilitated through the adoption of these technologies. HCPs in both primary care and tertiary care settings should suggest and support patients with implementing researched medication adherence mobile apps. The sample of this study included many patients with multiple chronic conditions. These participants appreciated the ability of the app to work across multiple chronic illnesses and its helpfulness when taking multiple medications. This finding underscores the importance of advocating for the use of medication adherence apps like Medisafe, which can work across a range of illnesses and medications and can be easily adjusted when medications change over the disease trajectory. Additionally, participants who used the adherence report felt that it provided positive reinforcement and was an incentive to reach higher levels of adherence. Similar to what has been reported in other studies, a majority of participants reported not using the refill reminder because they already received text alerts from their pharmacy, which they found helpful [18]. When HCPs select apps for patients to enhance their medication adherence, careful attention to app features and evaluation of existing research findings, such as the findings of this study, are important.

### Limitations

This study had several limitations. First, the study duration of 1 month does not provide insights regarding long-term patient satisfaction and continued use of app features, which are important aspects of chronic disease management. Though this study found high satisfaction and usability of the app during the first month of use, future studies should evaluate the role of time in app usage and satisfaction. Medisafe data and self-reported data showed that the uptake of educational information and the Medfriend social support feature was low. There was an unresolved discrepancy in the number of participants who reported accessing educational information and the actual usage identified from the Medisafe data. The discrepancy might be because Medisafe data only captured if the leaflet was accessed. Patients may have perceived accessing educational information as clicking the interaction button or clicking the “For You” tab at the bottom of the app, which Medisafe data did not capture. This can be clarified in the future by a more detailed definition of what constitutes the educational features of the app. Incorporating interviews to clarify subsequent survey results would strengthen future research studies. Another limitation of the study is that we did not gather participants’ inputs about why they chose not to use the Medfriend feature. Therefore, this study cannot speak about the potential benefits of this feature. Finally, although the app can be used in several languages and many patients who seek care at FQHCs speak a primary language other than English [48], the researchers were not able to incorporate multiple languages into the study protocol.

### Future Research

FQHCs and primary care settings working with adults who are chronically ill should consider medication adherence mobile phone apps as acceptable and practical tools to support medication adherence. Future studies could include a larger sample, consider the use of the available provider portal, and consider the experiences of both providers and patients. Cost analysis could be performed, and hospitalization rates and long-term usage and health outcomes over time could be studied. This study was for a 30-day period, but a study with a longer duration is necessary to see if the use of the app is sustained over time. In this study, only 2 participants reported barriers to using the app, and a larger long-term study could further explore barriers to sustained use and strategies for maintaining engagement in this population. Future research should use mixed methods to provide insights into app modification, the nature of barriers to use, and how app features, such as the Medfriend feature, could more easily be implemented among patients who might benefit the most from such features. As uptake of the additional features of the app, such as educational information and the Medfriend option, was low in this study, future research using larger datasets could explore what types of patients chose to use specific features and why they did. We purposefully did not require certain features to be used because we wanted to organically discover which features were most often used, if any.

Studying the usability of the app and its associated effects in ethnic populations in various languages is an important area of future research as community health centers serve a large number of patients with limited English proficiency [48].

### Conclusion

This study demonstrated that the medication adherence app is a useful, convenient, and feasible intervention in an FQHC setting. The various features of this app positively influenced the medication-taking behaviors of adults with one or more chronic illnesses. Participants were satisfied with the app and the features they chose to use. Reminders were viewed as helpful by the majority of participants. The medication list feature was particularly useful for patients who had multiple chronic conditions and saw multiple providers, and some used it to facilitate medication reconciliation. The findings of this study have important clinical implications, as clinicians can recommend the use of medication adherence apps as tools to provide support in adhering to medication regimens and as additional tools to use during medication reconciliation.

## Abbreviations

FQHC: federally qualified health center
HCP: health care provider
PI: principal investigator
RCT: randomized controlled trial

## Data Availability

Limited deidentified data are available upon request from the corresponding author.
